# From mechanism to clinical: research evolution and hotspot analysis of CD276/B7-H3 in cancer immunotherapy

**DOI:** 10.3389/fimmu.2026.1751400

**Published:** 2026-04-01

**Authors:** Chaojie Zhai, Jiarong Ye, Xiaoyan Li, Jipeng Liu, Xinchi Zhou, Zuao Wang, Xu Zhang, Wen Zeng, Hongliang Luo, Leifeng Chen, Fan Zhou

**Affiliations:** 1 Department of General Surgery, The Second Affiliated Hospital of Nanchang University, Nanchang, Jiangxi, China; 2 Precision Oncology Medicine Center, The Second Affiliated Hospital of Nanchang University, Nanchang, Jiangxi, China; 3 Biobank Center, The Second Affiliated Hospital of Nanchang University, Nanchang, Jiangxi, China; 4 Department of Anesthesiology, The Second Affiliated Hospital of Nanchang University, Nanchang, Jiangxi, China; 5 Department of Oncology, The Second Affiliated Hospital of Nanchang University, Nanchang, Jiangxi, China; 6 Medical Center for Cardiovascular Diseases, Neurological Diseases and Tumors of Jiangxi Province, The Second Affiliated Hospital of Nanchang University, Nanchang, China

**Keywords:** B7-H3, bibliometric analysis, cancer immunotherapy, CD276, immune checkpoint, research trends

## Abstract

**Background:**

CD276 (B7-H3) is a pivotal immune checkpoint molecule with dual roles in T-cell regulation and tumor immune evasion, representing a promising therapeutic target across multiple cancers. However, a comprehensive analysis of the research landscape, evolutionary pathways, and knowledge structure in this field remains lacking.

**Methods:**

Publications related to CD276/B7-H3 in cancer research were systematically retrieved from the Web of Science Core Collection (WoS, n = 688) and Scopus (n = 759) spanning from January 2001 to August 2025. After merging and de-duplication, a final corpus of 830 publications was analyzed using Bibliometrix, VOSviewer, CiteSpace, and Pajek to evaluate publication trends, influential authors and institutions, collaboration networks, core journals, co-citation patterns, and keyword evolution.

**Results:**

The field exhibited exponential growth with an annual publication increase of 21.77%. China and the United States were the dominant contributing countries. Journal for Immunotherapy of Cancer was the most productive journal, while Clinical Cancer Research produced the most impactful publications. Co-citation analysis highlighted foundational studies on B7-H3’s prognostic significance and recent breakthroughs in CAR T-cell therapy targeting B7-H3. Keyword evolution revealed a clear transition from early themes such as “expression” and “prognosis” to contemporary focuses including “immunotherapy,” “tumor microenvironment,” and “chimeric antigen receptor”.

**Conclusion:**

This study provides the first integrated bibliometric overview of CD276/B7-H3 research in cancer, illustrating a rapid transition from mechanistic exploration to clinical application. The findings underscore B7-H3’s importance as a pan-cancer antigen and immunotherapeutic target, offering valuable insights for guiding future research directions.

## 1 Introduction

Cancer immunotherapy has fundamentally transformed the landscape of oncology over the past decades, offering novel treatment paradigms that harness the body’s immune system to combat malignant cells (1–4). Among the most groundbreaking advances is immune checkpoint blockade (ICB), which targets inhibitory pathways to reinvigorate antitumor immunity (5–9). Antibodies directed against established immune checkpoints such as PD-1/PD-L1 and CTLA-4 have demonstrated remarkable clinical efficacy across a range of cancers (8, 10–14). However, a substantial proportion of patients exhibit innate or acquired resistance to these therapies, underscoring the urgent need to identify and characterize alternative immunomodulatory targets (5, 8, 15, 16).

CD276, commonly referred to as B7-H3, has emerged as a compelling player in the next generation of cancer immunotherapies (17–21). The human B7-H3 gene was first characterized by Sun et al., who identified a unique 4Ig-B7-H3 isoform resulting from gene duplication and alternative splicing (22). Initially discovered as a co-stimulatory molecule capable of enhancing T-cell activation and interferon-gamma production (23), it was subsequently found to also exert potent co-inhibitory functions, facilitating immune evasion in multiple tumor types (20, 24, 25). Studies by Prasad et al. first demonstrated that B7-H3 expressed on dendritic cells preferentially down-regulates Th1-mediated immune responses (26), while Castriconi et al. showed that 4Ig-B7-H3 serves as a tumor-associated antigen that protects neuroblastoma cells from NK cell-mediated lysis (27). This functional duality places B7-H3 at the center of a complex regulatory network within the tumor microenvironment (TME) (28–30). Its overexpression has been documented in a wide spectrum of malignancies—including carcinomas of the prostate, lung, breast, and kidneys—and is frequently correlated with advanced disease stage, metastatic progression, and unfavorable clinical outcomes (31–40). Early evidence of its prognostic value emerged from studies in gastric carcinoma (41) and prostate cancer (42), with subsequent work by Gregorio et al. demonstrating its independent prognostic significance in pediatric solid tumors such as neuroblastoma (43). These attributes nominally position B7-H3 not only as a prognostic biomarker but also as a promising therapeutic target.

The rapidly expanding corpus of literature on B7-H3 reflects growing interest in its biological roles and clinical potential. Research has extended from foundational studies elucidating its immunoregulatory mechanisms to translational efforts developing targeted interventions, such as monoclonal antibodies, antibody-drug conjugates (ADCs), and chimeric antigen receptor (CAR) T-cell therapies (19, 44–51). Despite this proliferation of publications, the vast and fragmented nature of the literature poses challenges for comprehensively assessing the intellectual architecture, collaborative networks, and knowledge dynamics within the field.

While narrative reviews have summarized biological aspects of B7-H3, a systematic, quantitative appraisal of the research landscape is currently lacking. Bibliometrics, a statistical approach to analyzing publication data, offers powerful tools to address this gap (52). By examining patterns of productivity, collaboration, citation, and keyword usage, bibliometric methods can map the evolution of a field, identify influential contributors and works, trace thematic shifts, and highlight emerging frontiers (52).

Therefore, this study employs a comprehensive bibliometric analysis to evaluate the global research output related to CD276/B7-H3 in cancer from 2001 to 2025. Our objectives are fourfold: (1) to quantify the growth trajectory and geographic distribution of publications; (2) to identify leading authors, institutions, and journals, along with their collaborative networks; (3) to delineate the intellectual foundation and thematic evolution through reference co-citation and keyword analysis; and (4) to map current research hotspots and suggest future directions. Through these analyses, we aim to provide an integrative perspective on the past, present, and potential future of B7-H3 research, thereby offering a valuable knowledge framework for researchers, clinicians, and policy-makers engaged in oncology and immuno-oncology.

## 2 Methods

Bibliometrics is a statistical method used to quantitatively analyze academic literature, aiming to map the cumulative scientific knowledge and evolutionary trends of a specific research field (53). It enables the assessment of productivity, impact, collaboration patterns, and thematic shifts through indicators such as publication counts, citation networks, co-authorship analysis, and keyword co-occurrence (52). This approach is particularly suitable for capturing the development trajectory of emerging research domains, such as CD276 in cancer, by providing a macroscopic overview of the scholarly landscape.

### 2.1 Sources of bibliometric data

**Figure 1:**
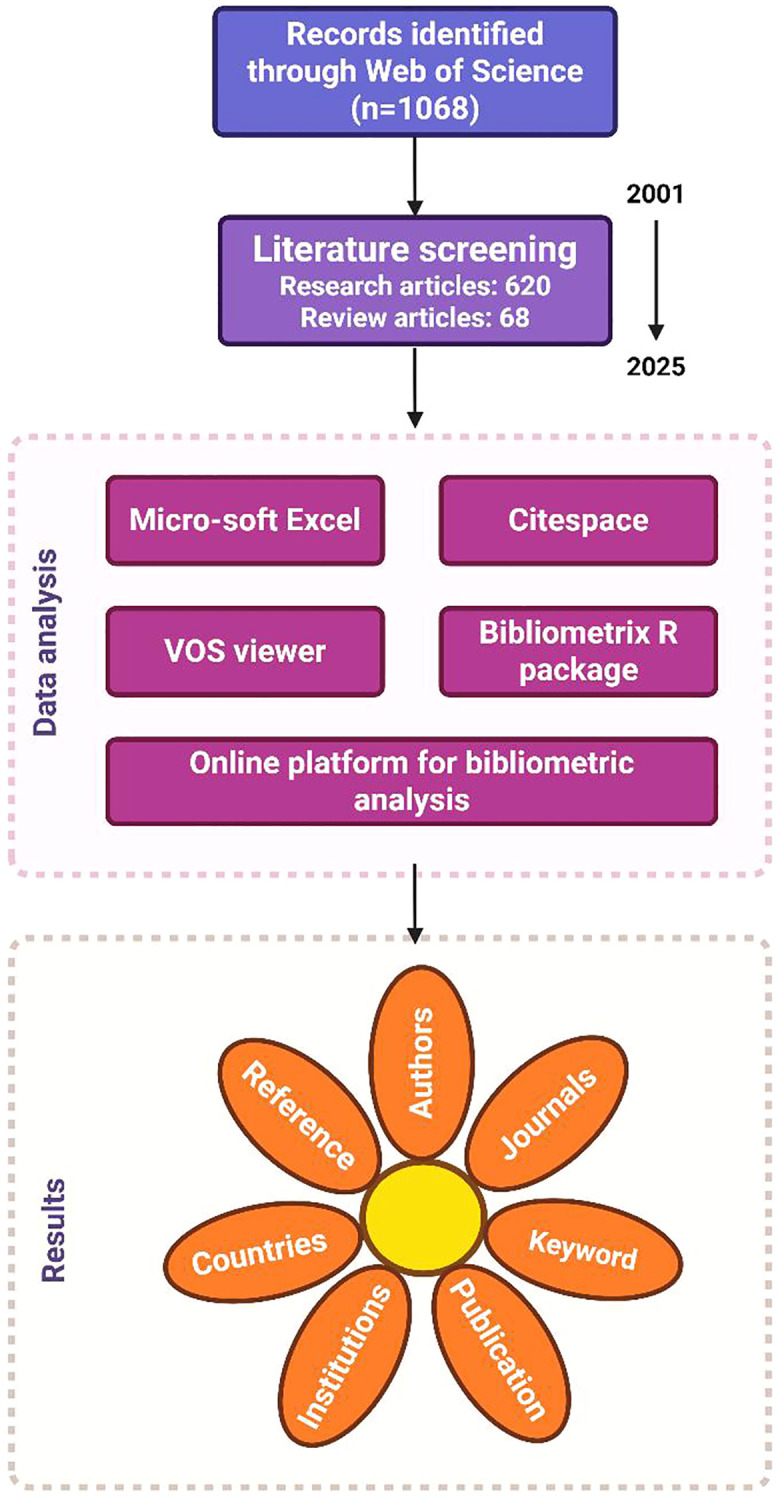
The process of screening articles.

A systematic search was conducted in both the Web of Science Core Collection and the Scopus databases to identify relevant studies. The retrieval period spanned from 1 January 2001 to 1 August 2025. The search strategy employed was as follows: (TI = ((“tumor” OR “cancer”) AND (“CD276” OR “B7-H3”OR “B7H3”)) OR AB = ((“tumor” OR “cancer”) AND (“CD276” OR “B7-H3”OR “B7H3”))) AND (DT=(“Article” OR “Review”)) The same strategy was adapted for the Scopus database using appropriate field identifiers. (**Figure 1**).

Although PubMed is a key biomedical database, it was not included as a data source in this study. This decision was based on methodological considerations specific to bibliometric analysis: Web of Science and Scopus provide comprehensive citation data and advanced analytical functionalities (e.g., co-citation analysis, standardized affiliation data) that are essential for constructing collaboration networks and knowledge structures. Additionally, the analytical tools employed (Bibliometrix, VOSviewer, CiteSpace) are optimized for data structures from these databases. While the exclusion of PubMed may omit some publications, the combined use of WoS and Scopus provides a sufficiently comprehensive foundation for mapping the global research landscape of CD276/B7-H3 in oncology.

### 2.2 Data screening, deduplication, and cleaning

To ensure data quality and consistency, a multi-step data processing protocol was implemented.

Screening: Two authors (CJZ and JRY) independently screened the titles and abstracts of all retrieved records to verify their relevance to CD276/B7-H3 in cancer research. Given the specificity of the search query, all records were deemed relevant, and no records were excluded at this stage. Any disagreements, if they had occurred, would have been resolved through discussion or consultation with a third reviewer (FZ).

Deduplication: Records from WoS and Scopus were merged into a single dataset. Duplicate publications were identified based on matching title, first author, last author, publication year, and digital object identifier (DOI) when available. If all these key fields matched exactly, the record was considered a duplicate and the entry with less complete metadata (or the later entry) was removed. For cases where some fields were missing (e.g., no DOI), a manual comparison was performed to confirm duplication. A total of 617 duplicates were identified and excluded, resulting in 830 unique publications for further analysis.

Institution name harmonization: To ensure accurate ranking and collaboration analysis, institutional affiliations were standardized. Variant spellings, abbreviations, and different representations of the same institution (e.g., “Peking University,” “Peking Univ,” “Beijing University”) were unified under a single preferred name (e.g., “Peking University”). Similarly, departmental affiliations (e.g., “Department of General Surgery, The Second Affiliated Hospital of Nanchang University”) were consolidated under the parent institution (“The Second Affiliated Hospital of Nanchang University”) to avoid fragmentation. This harmonization was performed using R software (version 4.3.2) with customized scripts for automated string matching and standardization, followed by manual verification by two authors (CJZ and JRY) to ensure accuracy.

### 2.3 Bibliometric analysis and visualization

Basic bibliometric analysis was conducted through data organization and preliminary calculations using Microsoft Excel 2021 (Microsoft Office, USA). For comprehensive scientometric mapping and analysis, the Bibliometrix R package (version 5.1.0) within R software (version 4.3.2) was employed to process and analyze the bibliographic records of the selected publications, in accordance with established methodologies (54). The Biblioshiny web application was used under the RStudio environment (version 2023.12.1 + 402; most recently accessed on 1 September 2025) to generate an interactive graphical interface. Furthermore, keyword co-occurrence analysis was performed with VOSviewer (version 1.6.20) to identify research hotspots related to CD276. To enhance structural interpretation of networks, additional analyses were carried out using CiteSpace (version 6.4.R1) and Pajek (version 6.01), which provided complementary perspectives on citation patterns and network visualization. (**Figure 1**).

## 3 Results

A total of 2,246 publications were initially retrieved, comprising 1,068 from Web of Science (WoS) and 1,178 from Scopus, published between 2001 and 2025. After filtering by document type and language, 688 records from WoS and 759 from Scopus met the inclusion criteria, all of which were either articles or reviews. These publications appeared in 279 (WoS) and 315 (Scopus) distinct journals, contributed by 3,507 authors in WoS and 3,917 in Scopus. The publications originated from 938 institutions across 34 countries/regions in WoS, with Scopus exhibiting similar geographic diversity.

The document types consisted of 620 research articles (90.11%) and 68 reviews (9.88%) in WoS, compared to 682 research articles (89.86%) and 77 reviews (10.14%) in Scopus. The average citation counts per document were 40.08 (WoS) and 38.10 (Scopus). Annual publication growth rates were 19.44% for WoS and 21.15% for Scopus. Each database contained only two single-authored publications. International collaboration rates were 16.01% (WoS) and 17.39% (Scopus), with a mean co-author count of 9.82 and 9.87 per document, respectively. A total of 1,207 and 1,405 unique author keywords were identified in WoS and Scopus. The average document age was 4.87 years (WoS) and 4.77 years (Scopus).

After merging both databases and removing 617 duplicates, 830 unique publications were retained for analysis. This consolidated corpus consisted of 740 research articles (89.16%) and 90 reviews (10.84%), published across 338 sources, involving 4,218 authors and 1,487 unique author keywords. Only two documents were single-authored. The international co-authorship rate was 13.25%, with an average of 9.83 co-authors per document. The average citation count was 36.49, and the mean document age was 4.64 years. The overall annual growth rate of publications was 21.77%.

### 3.1 Growth trend of publications

**Figure 2:**
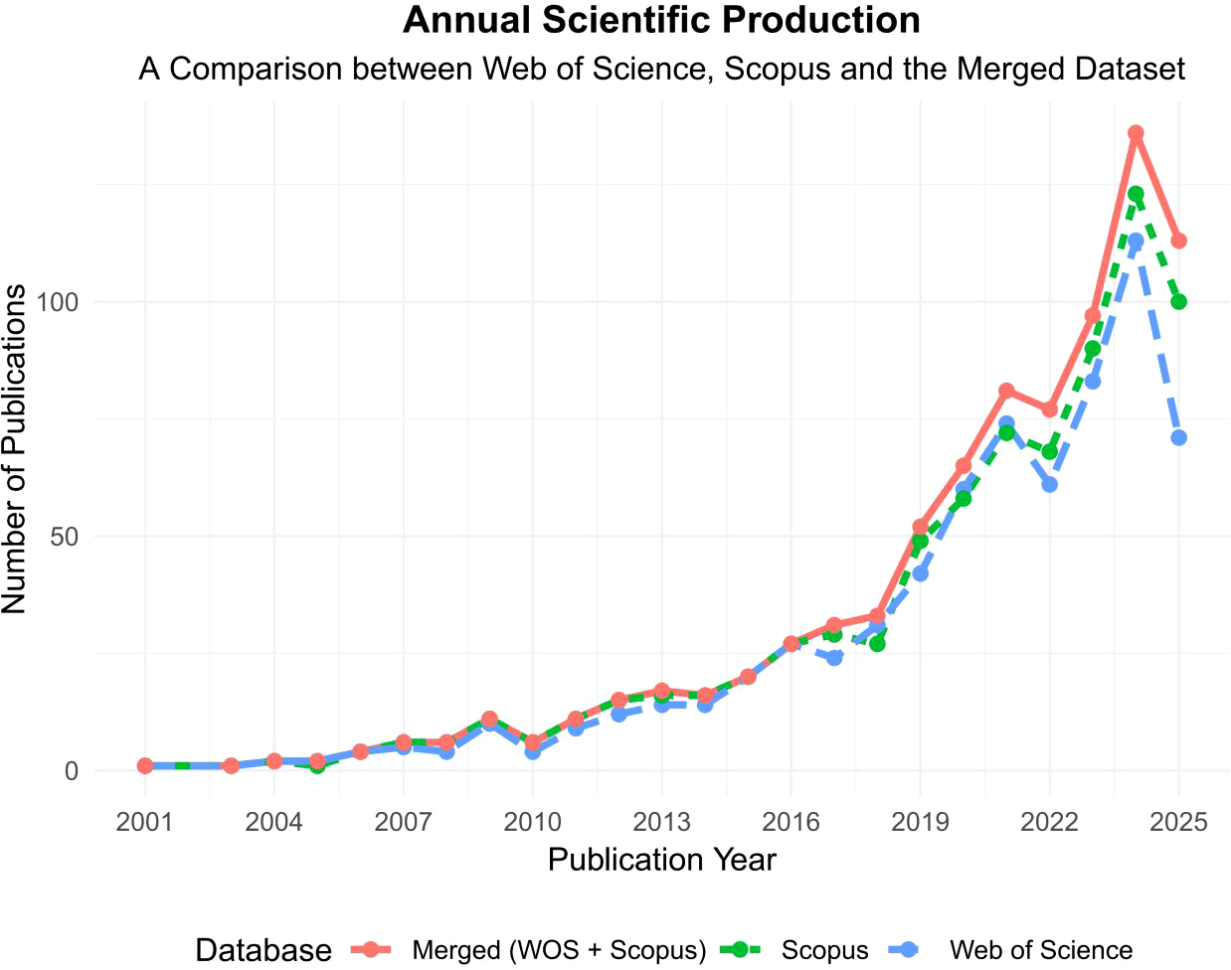
Annual publication trends on CD276/B7-H3 research.

As shown in **Figure 2**, the annual scientific production related to CD276/B7-H3 in cancer research exhibited a consistent upward trend across both Web of Science and Scopus from 2001 to 2025, with the merged dataset providing the most comprehensive coverage and confirming the robustness of this growth, emphasizing the molecule’s increasing importance as a compelling immunotherapeutic target and mirroring the broader shift in immuno-oncology beyond canonical checkpoints like PD-1 and CTLA-4.

### 3.2 Countries or regions

**Figure 3:**
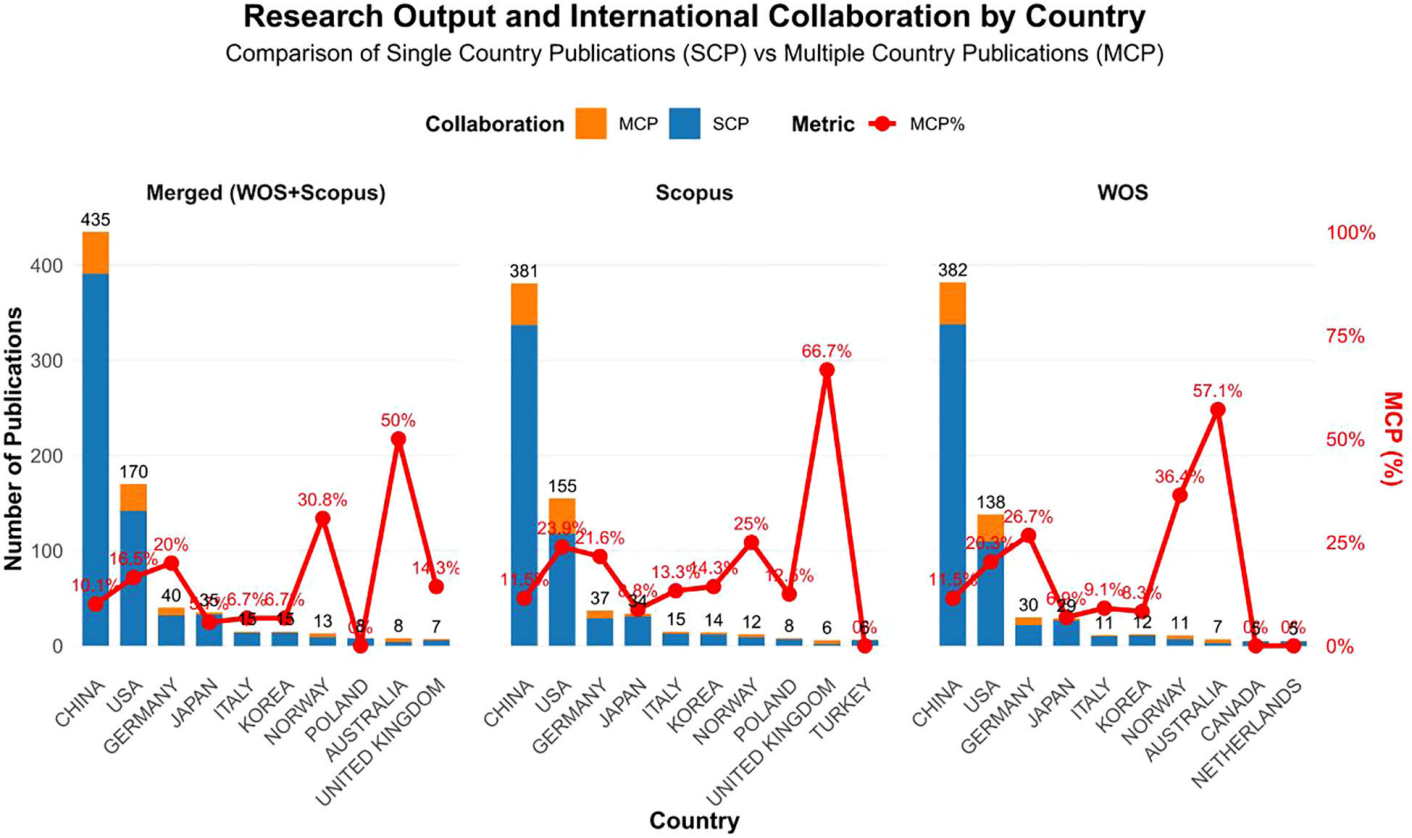
Bar charts of the top 10 most productive countries for SCP and MCP of corresponding author’s countries.

**Figure 3** and **Table 1** present the top 10 most productive countries/regions in CD276/B7-H3 cancer research across the merged, WoS, and Scopus datasets. China led in publication output across all three datasets, accounting for 52.4% (435 articles) of the merged corpus, though it demonstrated a relatively low rate of international collaboration (MCP% = 10.1%). The United States ranked second (20.5%, 170 articles), with a moderate collaboration rate (16.5% MCP). Notably, several countries with lower output displayed high international collaboration rates, such as Australia (50% MCP) and Norway (30.8% MCP) in the merged dataset. The consistent patterns across databases confirm the dominance of China and the U.S., with significant collaborative involvement from Germany (40 articles), Japan (35 articles), Italy (15 articles), and South Korea (15 articles).

**Table 1:** The top 10 most productive countries.

Database	Country	Articles	Articles %	SCP	MCP	MCP %
Merged (WOS+Scopus)	CHINA	435	52.4	391	44	10.1
	USA	170	20.5	142	28	16.5
	GERMANY	40	4.8	32	8	20
	JAPAN	35	4.2	33	2	5.7
	ITALY	15	1.8	14	1	6.7
	KOREA	15	1.8	14	1	6.7
	NORWAY	13	1.6	9	4	30.8
	AUSTRALIA	8	1	4	4	50
	POLAND	8	1	8	0	0
	UNITED KINGDOM	7	0.8	6	1	14.3
WOS	CHINA	382	55.5	338	44	11.5
	USA	138	20.1	110	28	20.3
	GERMANY	30	4.4	22	8	26.7
	JAPAN	29	4.2	27	2	6.9
	KOREA	12	1.7	11	1	8.3
	ITALY	11	1.6	10	1	9.1
	NORWAY	11	1.6	7	4	36.4
	AUSTRALIA	7	1	3	4	57.1
	CANADA	5	0.7	5	0	0
	NETHERLANDS	5	0.7	5	0	0
Scopus	CHINA	381	50.2	337	44	11.5
	USA	155	20.4	118	37	23.9
	GERMANY	37	4.9	29	8	21.6
	JAPAN	34	4.5	31	3	8.8
	ITALY	15	2	13	2	13.3
	KOREA	14	1.8	12	2	14.3
	NORWAY	12	1.6	9	3	25
	POLAND	8	1.1	7	1	12.5
	TURKEY	6	0.8	6	0	0
	UNITED KINGDOM	6	0.8	2	4	66.7

**Figure 4:**
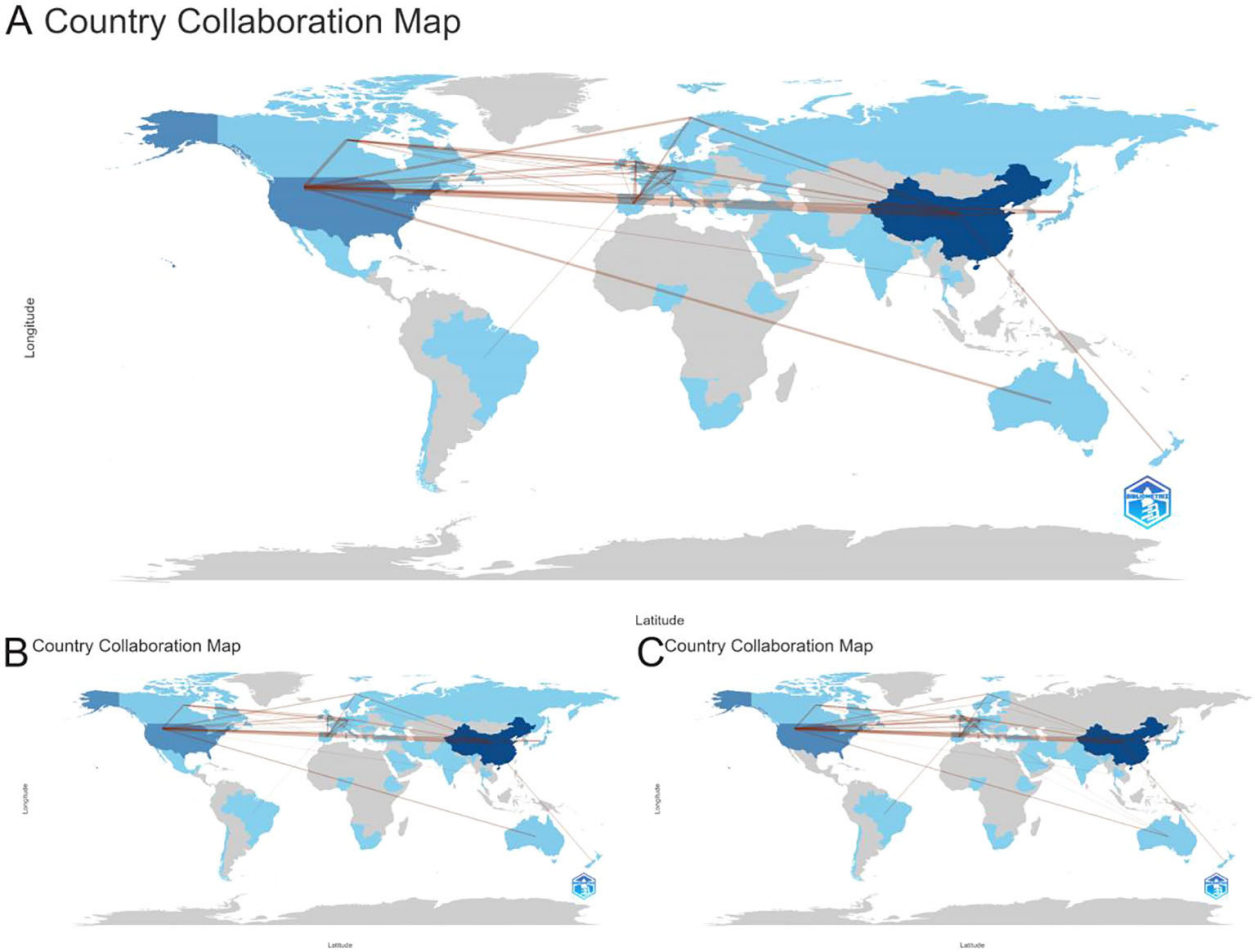
International collaboration networks in CD276/B7-H3 cancer research. **(A)** Collaboration map based on the merged dataset of WOS and Scopus. **(B)** Collaboration network derived from WOS. **(C)** Collaboration network derived from Scopus. Nodes represent countries, with thicker edges denoting stronger collaborative ties.

Moreover, cooperation network analysis across all three datasets consistently identified the most frequent and robust collaborative partnership to be between China and the United States. This central bilateral relationship underscores a key axis of international collaboration within the global research landscape of CD276/B7-H3 cancer research (**Figure 4**). The persistence of this partnership across WOS, Scopus, and the merged corpus highlights its foundational role in shaping the field’s transnational knowledge production.

Nodes represent countries, and connecting edges indicate co-authorship relationships, with thicker edges denoting stronger collaborative ties. The consistent prominence of certain partnerships—particularly between China and the United States—across all three panels highlights key cooperative axes in the global research landscape.

Furthermore, analysis of the top 10 countries by citations (**Table 2**) reveals distinct patterns of research impact in CD276/B7-H3 cancer studies. The United States led in average citations per article (AC: 66.00–76.90), indicating high influence despite a lower output than China. China ranked first in total publications but had moderate average citations (AC: 26.80–28.50). Several European nations demonstrated exceptional impact with limited output; France achieved notably high average citations (e.g., 415.70 in WOS), and Sweden and Norway also maintained high AC values. Japan, Germany, Italy, and South Korea consistently appeared among the top contributors. The concordance between WOS and Scopus underscores the reliability of these trends, confirming that the U.S. (AC: 66.0), France (AC: 311.8), Norway (AC: 58.6), and Sweden (AC: 62.0) produce particularly influential research within this domain.

**Table 2:** Ranking of the top 10 countries/regions by total citations. USA, the United States of America; TC, total citations, AC, average citations.

Database	Country	AC	TC	Average article citations
Merged	CHINA	435	11661	26.80
	USA	170	11217	66.00
	JAPAN	35	1252	35.80
	FRANCE	4	1247	311.80
	GERMANY	40	926	23.10
	NORWAY	13	762	58.60
	ITALY	15	621	41.40
	SWEDEN	5	310	62.00
	KOREA	15	286	19.10
	UNITED KINGDOM	7	257	36.70
WOS	CHINA	382	10876	28.50
	USA	138	10607	76.90
	FRANCE	3	1247	415.70
	JAPAN	19	1070	36.90
	GERMANY	30	760	25.30
	NORWAY	11	714	64.90
	ITALY	11	444	40.40
	SWEDEN	1	292	292.00
	KOREA	12	235	19.60
	AUSTRALIA	7	180	25.70
Scopus	USA	155	11213	72.30
	CHINA	382	10874	28.50
	JAPAN	34	1322	38.90
	GERMANY	37	915	24.70
	NORWAY	12	667	55.60
	ITALY	15	659	43.90
	SWEDEN	5	325	65.00
	UNITED KINGDOM	6	245	40.80
	KOREA	14	215	15.40
	BELGIUM	5	148	29.60

### 3.3 Institutions

**Table 3** presents the top 10 most productive institutions in CD276/B7-H3 research. Soochow University was the most prolific across all datasets (91 articles in Merged), though with moderate average citations (AC: 31.64). In contrast, the University of Oslo, while ranking second in output (60 articles), achieved notably higher impact with the highest total citations (TC: 3279) and a strong average citation rate (AC: 54.65).

**Table 3:** Numbers of article and citation of top 10 affiliation.

Database	Affiliation	NP	TC	AC
Merged	SOOCHOW UNIVERSITY	91	2879	31.64
	UNIVERSITY OF OSLO	60	3279	54.65
	PEKING UNIVERSITY	50	970	19.40
	XUZHOU MEDICAL UNIVERSITY	36	298	8.28
	NANJING MEDICAL UNIVERSITY	35	384	10.97
	JIANGNAN UNIVERSITY	34	878	25.82
	HARVARD UNIVERSITY	33	1495	45.30
	UNIVERSITY OF GENOA	32	1835	57.34
	HUAZHONG UNIVERSITY OF SCIENCE AND TECHNOLOGY	28	264	9.43
	NATIONAL INSTITUTES OF HEALTH	28	1580	56.43
WOS	SOOCHOW UNIVERSITY	81	2697	33.30
	UNIVERSITY OF OSLO	59	3237	54.86
	PEKING UNIVERSITY	45	922	20.49
	XUZHOU MEDICAL UNIVERSITY	36	298	8.28
	NANJING MEDICAL UNIVERSITY	35	384	10.97
	JIANGNAN UNIVERSITY	33	878	26.61
	UNIVERSITY OF GENOA	30	1655	55.17
	HARVARD UNIVERSITY	29	1478	50.97
	HEBEI MEDICAL UNIVERSITY	23	1354	58.87
	NATIONAL INSTITUTES OF HEALTH	22	1520	69.09
Scopus	SOOCHOW UNIVERSITY	88	2771	31.49
	PEKING UNIVERSITY	36	841	23.36
	HARVARD UNIVERSITY	30	1630	54.33
	SICHUAN UNIVERSITY	28	1074	38.36
	NATIONAL INSTITUTES OF HEALTH	27	910	33.70
	ALBERT EINSTEIN COLLEGE OF MEDICINE	25	2111	84.44
	JIANGNAN UNIVERSITY	24	805	33.54
	HUAZHONG UNIVERSITY OF SCIENCE AND TECHNOLOGY	22	381	17.32
	UNIVERSITY OF OSLO	18	1129	62.72
	UNIVERSITY OF TUEBINGEN	18	166	9.22

Several institutions with focused output demonstrated high influence, including the University of Genoa (AC: 57.34), Harvard University (AC: 45.30), and the National Institutes of Health (AC: 56.43). Albert Einstein College of Medicine excelled in Scopus with an outstanding AC of 84.44. Chinese institutions such as Peking University ranked highly in productivity but exhibited comparatively lower average citation rates. The consistent rankings between WOS and Scopus validate these trends, with the merged dataset providing a comprehensive overview of core contributing institutions.

**Figure 5:**
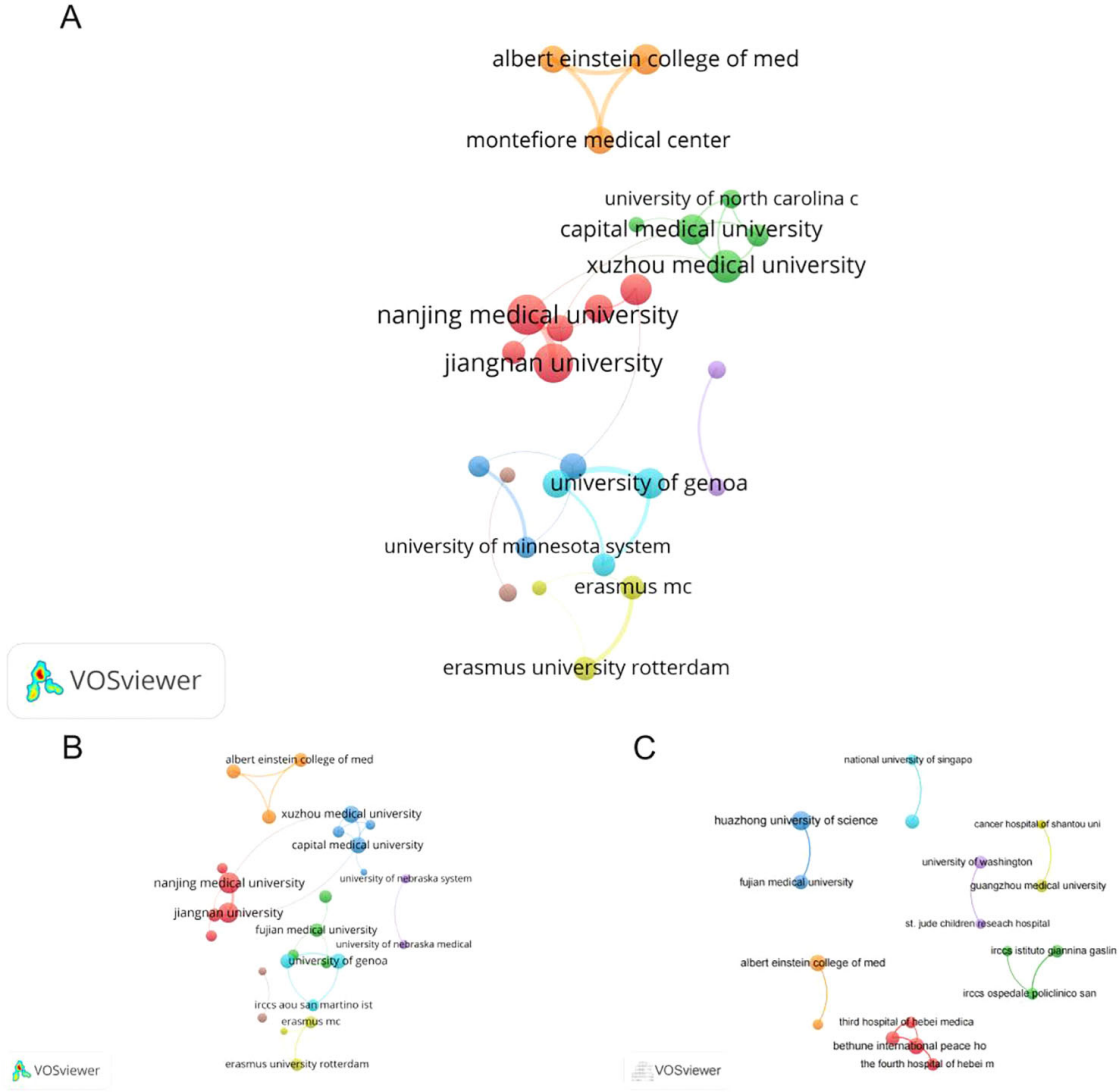
Institutional collaboration networks in CD276/B7-H3 cancer research. **(A)** Network derived from the merged dataset of WoS and Scopus. **(B)** Network based on WoS. **(C)** Network based on Scopus. Nodes represent institutions (e.g., universities or research centers), with node size proportional to the total number of publications from that institution. Connecting lines indicate collaborative relationships, and line thickness corresponds to the strength of collaboration (i.e., number of co-authored publications).

Institutional collaboration networks across the three datasets reveal distinct research communities focused on CD276/B7-H3 (**Figure 5**). Chinese institutions form the core of multiple clusters, with Nanjing Medical University, Jiangnan University, and the Chinese Academy of Sciences serving as central hubs, exhibiting high betweenness centrality in both the merged and WOS datasets. Capital Medical University also played a key bridging role.

A consistent European cluster centered around the University of Genoa and its affiliated IRCCS institutes appeared across all databases, indicating stable international collaboration. U.S. institutions such as the University of North Carolina and Albert Einstein College of Medicine also participated in cross-border partnerships, particularly with Chinese universities.

While many clusters remain nationally oriented—especially within China—several groups demonstrate active international cooperation. The structural consistency across WOS, Scopus, and the merged dataset confirms the robustness of these collaborative patterns, with Chinese institutions (e.g., Nanjing Medical University, Jiangnan University) dominating in structural importance and European (e.g., University of Genoa) and U.S. (e.g., University of North Carolina) groups facilitating global knowledge exchange.

In each panel, nodes represent institutions (e.g., universities or research centers), with node size proportional to the total number of publications from that institution. Connecting lines indicate collaborative relationships, and line thickness corresponds to the strength of collaboration (i.e., number of co-authored publications).

### 3.4 Authors

To validate the conformity of the combined data to classical bibliometric patterns, Lotka’s Law of scientific productivity was applied to Web of Science. The resulting distributions consistently adhered to the expected pattern across databases, confirming the suitability of the data for further bibliometric analysis (**Supplementary Figure 1**) (55). We present the top 10 most prolific authors based on publication count in **Table 4**. Among these, ZHANG XG and ZHANG GB were the most productive, with 32 and 31 publications, respectively. They also exhibited high citation impact, with total citations (TC) of 1582 and 1603, and average citations per publication (AC) of 49.44 and 51.71. Notably, FERRONE S and FODSTAD O demonstrated particularly high average citations per paper (AC = 74.19 and 77.73, respectively), reflecting the significant influence of their work. Additionally, TONG AP, despite a relatively recent entry into the field (PY_start = 2019), has achieved a high m_index of 1.714, indicating a rapid accumulation of influence relative to time active in the field. The data illustrate not only variations in productivity among leading authors but also differences in citation impact and the tempo of academic influence, providing a nuanced overview of key contributors in this research domain.

**Table 4:** The top 10 most prolific authors based on publication count.

Author	NP	TC	AC	h_index	g_index	m_index	PY_start
ZHANG XG	32	1582	49.44	20	32	0.952	2005
ZHANG GB	31	1603	51.71	22	31	1.048	2005
WANG Y	24	731	30.46	12	24	0.706	2009
ZHANG Y	20	342	17.10	9	18	1	2017
WANG L	17	796	46.82	15	17	1.154	2013
FERRONE S	16	1187	74.19	12	16	0.857	2012
TONG AP	16	559	34.94	12	16	1.714	2019
FODSTAD O	15	1166	77.73	14	15	0.778	2008
WANG J	14	477	34.07	12	14	0.923	2013
HUA D	14	521	37.21	11	14	0.846	2013

NP, Number of Publications; PY_start:, Publication Year start.

**Figure 6:**
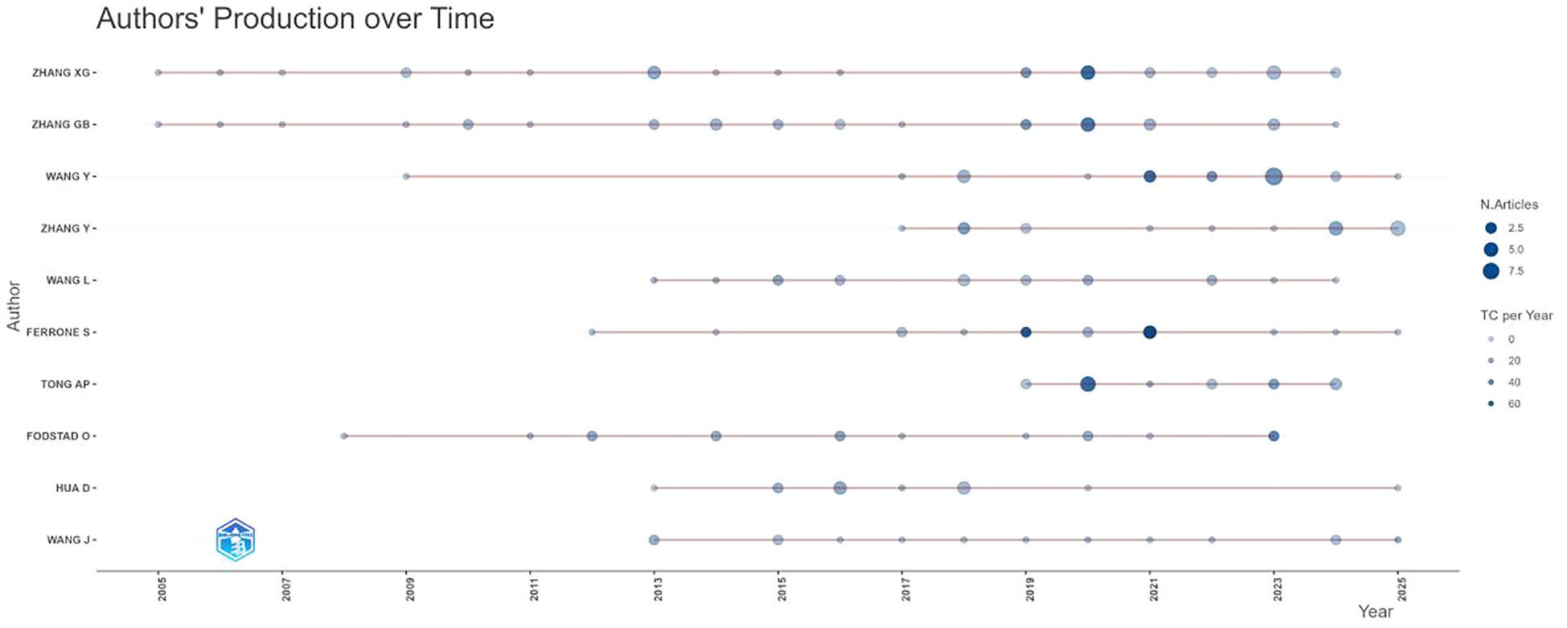
Authors’ production and citation over time.

The annual publication output and corresponding citation impact of the top productive authors over time is illustrated in **Figure 6**. As shown, authors such as ZHANG XG and ZHANG GB demonstrate sustained scholarly activity throughout the observed period, with notable publication volumes beginning around 2005 and continuing through subsequent years. Their works have accumulated significant citations, reflecting continued influence in the field. In contrast, some researchers, including TONG AP and FODSTAD O, show more concentrated periods of productivity, particularly in recent years, suggesting emerging or intensified research engagement. The temporal distribution of publications across major authors highlights both established and rising contributors, while the trend in total citations (TC) per year underscores the growing recognition and impact of their work within the academic community. This chronological overview not only maps the active involvement of key researchers but also suggests shifting emphases and potential evolution of research hotspots over time.

**Figure 7:**
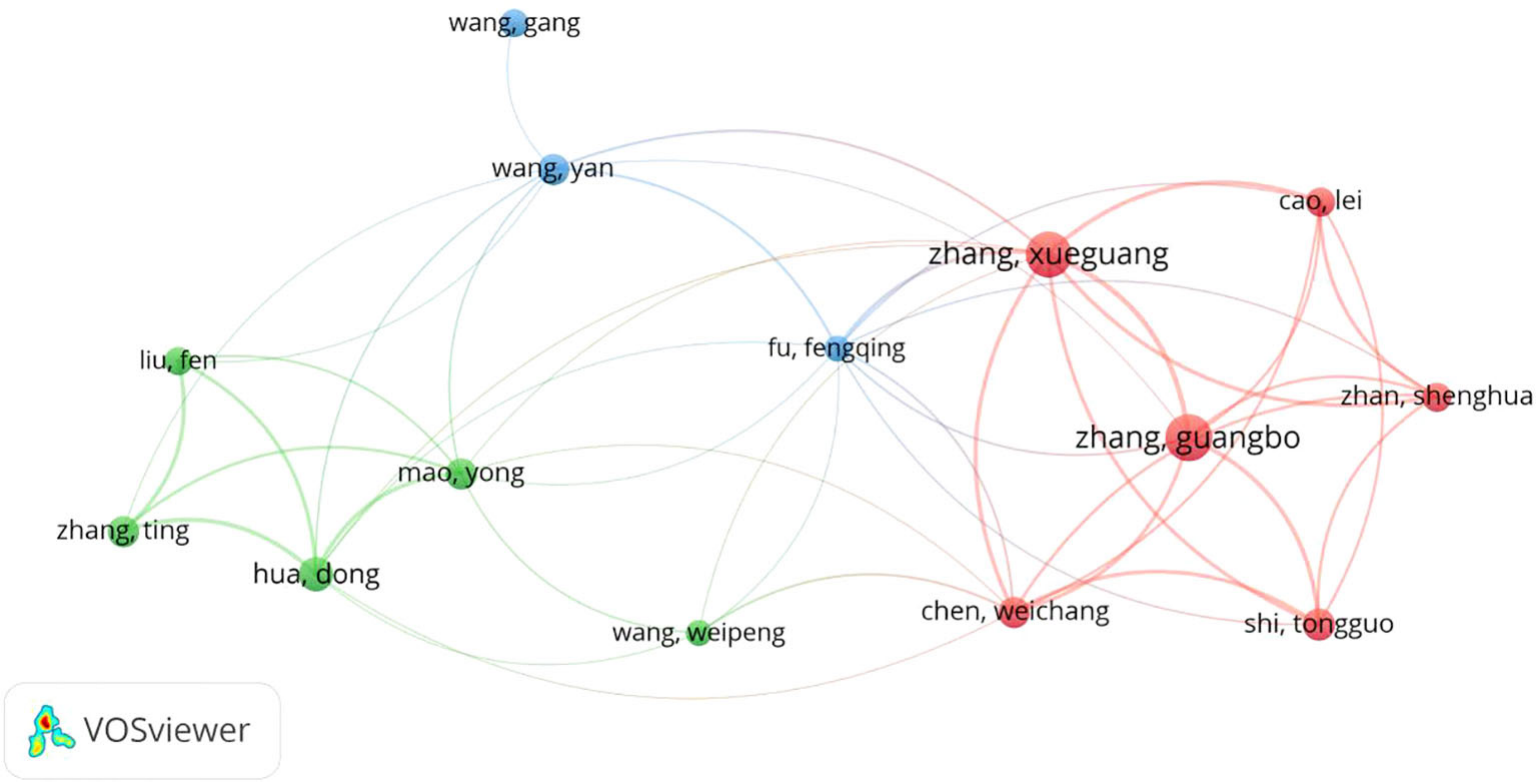
The cooperation relationships of authors. Each node represents an author; its size corresponds to the author’s number of publications. Lines between nodes indicate co-authorship, with thicker lines denoting stronger collaborative ties (i.e., more co-authored papers). Authors are color-coded into clusters based on the density of their collaborative links, highlighting distinct research communities.

**Figure 7** reveals a prominent collaborative cluster centered around WANG G and WANG Y, who appear to serve as central hubs connecting multiple research groups. This core group maintains strong collaborations with other productive investigators including ZHANG XG and FU FQ, suggesting these researchers may form the backbone of one or more major research teams in the CD276/B7-H3 field.

Additional significant contributors such as ZHANG GB, CHEN WC, and SHI TG are also well-integrated within the network, indicating robust interdisciplinary or inter-institutional cooperation. The presence of several other researchers including LIU F, ZHANG T, HUA D, MAO Y, and WANG WP further demonstrates the expansion of this collaborative network.

The overall structure suggests that CD276/B7-H3 research is conducted through relatively tightly-knit collaborative teams rather than isolated individual efforts. This pattern of cooperation likely enhances knowledge sharing, resource pooling, and research productivity, potentially accelerating scientific output in this field. The interconnected nature of these collaborations may also facilitate the translation of basic findings into clinical applications through multidisciplinary approaches.

Future studies could further investigate the institutional affiliations and geographical distribution of these authors to better understand the dynamics of domestic and international collaborations in CD276/B7-H3 research.

### 3.5 Journals

Based on Bradford’s Law, the distribution of core sources in the research domain is illustrated in **Supplementary Figure 2** (56). Analysis of the top publishing journals reveals a stable core of key outlets for CD276/B7-H3 research across all three databases (**Table 5**). Frontiers in Immunology leads in publication volume in the merged dataset (NP = 28), while Clinical Cancer Research stands out as the most influential journal, boasting the highest total and average citations (TC = 2501; AC = 125.05 in merged), underscoring its significant impact despite a lower article output.

**Table 5:** The top 10 journals in terms of number of publications.

Database	Source	h_index	g_index	m_index	TC	NP	AC	PY_start
Merged	FRONTIERS IN IMMUNOLOGY	12	27	1	758	28	27.07	2014
	JOURNAL FOR IMMUNOTHERAPY OF CANCER	10	21	1.429	466	22	21.18	2019
	FRONTIERS IN ONCOLOGY	10	20	1.25	402	21	19.14	2018
	CLINICAL CANCER RESEARCH	16	20	0.889	2501	20	125.05	2008
	CANCERS	9	14	1.125	216	18	12.00	2018
	ONCOTARGET	14	14	1.273	630	14	45.00	2015
	CANCER IMMUNOLOGY IMMUNOTHERAPY	8	13	0.5	424	13	32.62	2010
	INTERNATIONAL JOURNAL OF MOLECULAR SCIENCES	8	13	1.333	192	13	14.77	2020
	SCIENTIFIC REPORTS	8	13	0.8	247	13	19.00	2016
	ONCOTARGETS AND THERAPY	12	12	0.923	488	12	40.67	2013
WOS	JOURNAL FOR IMMUNOTHERAPY OF CANCER	10	21	1.429	466	21	22.19	2019
	FRONTIERS IN IMMUNOLOGY	11	20	0.917	716	20	35.80	2014
	FRONTIERS IN ONCOLOGY	9	17	1.125	373	17	21.94	2018
	CLINICAL CANCER RESEARCH	16	16	0.889	2246	16	140.38	2008
	CANCER IMMUNOLOGY IMMUNOTHERAPY	8	13	0.5	424	13	32.62	2010
	CANCERS	8	13	1	188	13	14.46	2018
	SCIENTIFIC REPORTS	7	12	0.7	236	12	19.67	2016
	ONCOTARGET	11	11	1	563	11	51.18	2015
	INTERNATIONAL JOURNAL OF MOLECULAR SCIENCES	6	11	1	148	11	13.45	2020
	ONCOTARGETS AND THERAPY	10	10	0.769	390	10	39.00	2013
Scopus	FRONTIERS IN IMMUNOLOGY	12	25	1	797	25	31.88	2014
	JOURNAL FOR IMMUNOTHERAPY OF CANCER	10	21	1.429	522	21	24.86	2019
	CLINICAL CANCER RESEARCH	16	20	0.889	2647	20	132.35	2008
	FRONTIERS IN ONCOLOGY	10	19	1.25	410	19	21.58	2018
	CANCER IMMUNOLOGY, IMMUNOTHERAPY	9	15	0.563	500	15	33.33	2010
	CANCERS	8	14	1	218	15	14.53	2018
	ONCOTARGET	14	14	1.273	654	14	46.71	2015
	INTERNATIONAL JOURNAL OF MOLECULAR SCIENCES	8	13	1.333	199	13	15.31	2020
	ONCOTARGETS AND THERAPY	12	12	0.923	515	12	42.92	2013
	ONCOLOGY LETTERS	10	12	0.769	378	12	31.50	2013

Notably, journals such as Journal for Immunotherapy of Cancer and Frontiers in Oncology consistently rank highly, reflecting the field’s strong focus on immunotherapy and translational cancer research. The presence of journals with high m-index values (e.g., J Immunother Cancer, m-index=1.429) indicates that several influential sources have emerged recently, contributing actively to the evolving discourse. This consolidated group of journals, comprising both established high-impact titles and newer open-access platforms, serves as the primary dissemination channel for research in this field.

**Figure 8:**
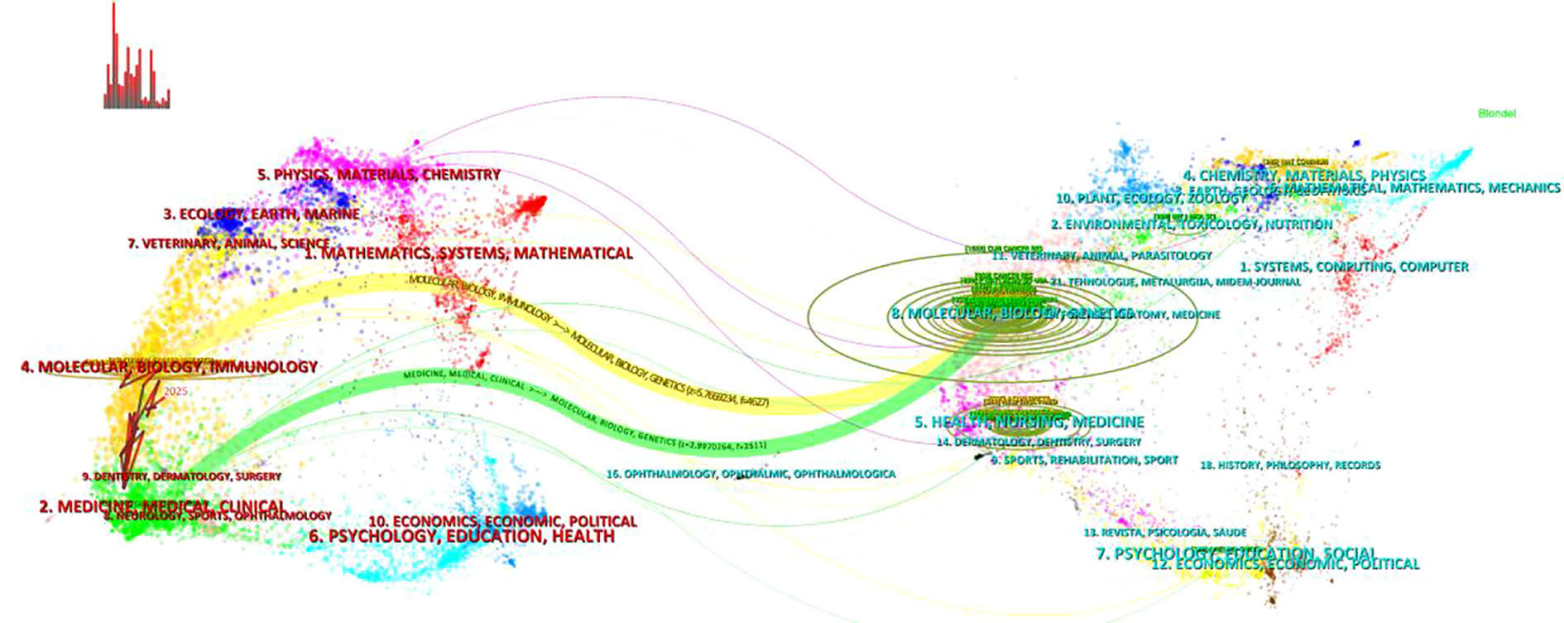
The dual-map overlay of journals of CD276 in cancer field. The left section displays the disciplinary distribution of citing journals, reflecting current research foci. On the right, clusters of cited journals represent the foundational knowledge base. The curvature between them illustrates citation relationships and knowledge flow, with circle sizes corresponding to the volume of publications in each discipline.

According to the dual-map overlay of journals based on Web of Science data (**Figure 8**), the intellectual structure and research trends of CD276 in cancer can be visually interpreted. The left side of the map, representing citing journals, reflects current research focus and is concentrated in Molecular, Biological, and Immunological sciences, as well as Health-related fields. The right side, corresponding to cited journals, forms the foundational knowledge base, with strong clusters in Medicine, Medical, Clinical, and Psychological/Educational/Health research.

Multiple citation paths indicate significant knowledge flow from foundational disciplines—particularly psychological, educational, and multidisciplinary health journals—toward current applied research in molecular and clinical oncology. This pattern underscores the highly interdisciplinary nature of CD276 research, integrating not only biomedical fundamentals but also insights from broader health and psychological sciences. The overlay demonstrates that CD276 investigations are firmly rooted in established medical literature while actively advancing translational research, illustrating a dynamic and evolving scholarly exchange across diverse domains.

### 3.6 Top articles

**Table 6** presents the top 10 most cited references on CD276/B7-H3 in cancer, derived from Web of Science and Scopus. The foundational work by Chapoval et al. (2001) remains the most cited, establishing B7-H3’s role in T-cell activation. Recent studies reflect a shift toward immunotherapy, with significant attention to CAR-T cell research by Majzner et al. (2019) and Du et al. (2019). Gill et al. (2021) exhibits the highest annual citation rate, indicating growing interest in clinical applications. Earlier articles by Zang et al. (2007) and Sun et al. (2006) continue to be influential, underscoring lasting interest in B7-H3’s clinical implications. Overall, the citation landscape traces the evolution from mechanistic discovery to translational immunotherapies.

**Table 6:** The top 10 most cited references related to CD276 research in the cancer field.

Database	Author, year	TI	TC	TC per year
WOS	CHAPOVAL, 2001	B7-H3:: A COSTIMULATORY MOLECULE FOR T CELL ACTIVATION AND IFN-Γ PRODUCTION	857	34.3
	GILL, 2021	ADVANCING THERAPY FOR OSTEOSARCOMA	563	112.6
	MAJZNER, 2019	CAR T CELLS TARGETING B7-H3, A PAN-CANCER ANTIGEN, DEMONSTRATE POTENT PRECLINICAL ACTIVITY AGAINST PEDIATRIC SOLID TUMORS AND BRAIN TUMORS	455	65
	PICARDA, 2016	MOLECULAR PATHWAYS: TARGETING B7-H3 (CD276) FOR HUMAN CANCER IMMUNOTHERAPY	406	40.6
	ANDREWS, 2019	INHIBITORY RECEPTORS AND LIGANDS BEYOND PD-1, PD-L1 AND CTLA-4: BREAKTHROUGHS OR BACKUPS	401	57.3
	LEHMANN, 2008	SENESCENCE-ASSOCIATED EXOSOME RELEASE FROM HUMAN PROSTATE CANCER CELLS	381	21.2
	DU, 2019	ANTITUMOR RESPONSES IN THE ABSENCE OF TOXICITY IN SOLID TUMORS BY TARGETING B7-H3 VIA CHIMERIC ANTIGEN RECEPTOR T CELLS	340	48.6
	ZANG, 2007	B7-H3 AND B7X ARE HIGHLY EXPRESSED IN HUMAN PROSTATE CANCER AND ASSOCIATED WITH DISEASE SPREAD AND POOR OUTCOME	326	17.2
	SEAMAN, 2017	ERADICATION OF TUMORS THROUGH SIMULTANEOUS ABLATION OF CD276/B7-H3-POSITIVE TUMOR CELLS AND TUMOR VASCULATURE	305	33.9
	SUN, 2006	B7-H3 AND B7-H4 EXPRESSION IN NON-SMALL-CELL LUNG CANCER	292	14.6
Scopus	CHAPOVAL, 2001	B7-H3: A COSTIMULATORY MOLECULE FOR T CELL ACTIVATION AND IFN-Γ PRODUCTION	915	36.6
	GILL, 2021	ADVANCING THERAPY FOR OSTEOSARCOMA	588	117.6
	MAJZNER, 2019	CAR T CELLS TARGETING B7-H3, A PAN-CANCER ANTIGEN, DEMONSTRATE POTENT PRECLINICAL ACTIVITY AGAINST PEDIATRIC SOLID TUMORS AND BRAIN TUMORS	489	69.9
	PICARDA, 2016	MOLECULAR PATHWAYS: TARGETING B7-H3 (CD276) FOR HUMAN CANCER IMMUNOTHERAPY	433	43.3
	LEHMANN, 2008	SENESCENCE-ASSOCIATED EXOSOME RELEASE FROM HUMAN PROSTATE CANCER CELLS	406	22.6
	DU, 2019	ANTITUMOR RESPONSES IN THE ABSENCE OF TOXICITY IN SOLID TUMORS BY TARGETING B7-H3 VIA CHIMERIC ANTIGEN RECEPTOR T CELLS	361	51.6
	ZANG, 2007	B7-H3 AND B7X ARE HIGHLY EXPRESSED IN HUMAN PROSTATE CANCER AND ASSOCIATED WITH DISEASE SPREAD AND POOR OUTCOME	350	18.4
	SEAMAN, 2017	ERADICATION OF TUMORS THROUGH SIMULTANEOUS ABLATION OF CD276/B7-H3-POSITIVE TUMOR CELLS AND TUMOR VASCULATURE	323	35.9
	SUN, 2006	B7-H3 AND B7-H4 EXPRESSION IN NON-SMALL-CELL LUNG CANCER	307	15.3
	KHAN, 2020	NK CELL-BASED IMMUNE CHECKPOINT INHIBITION	287	47.8

TI, Title.

**Figure 9:**
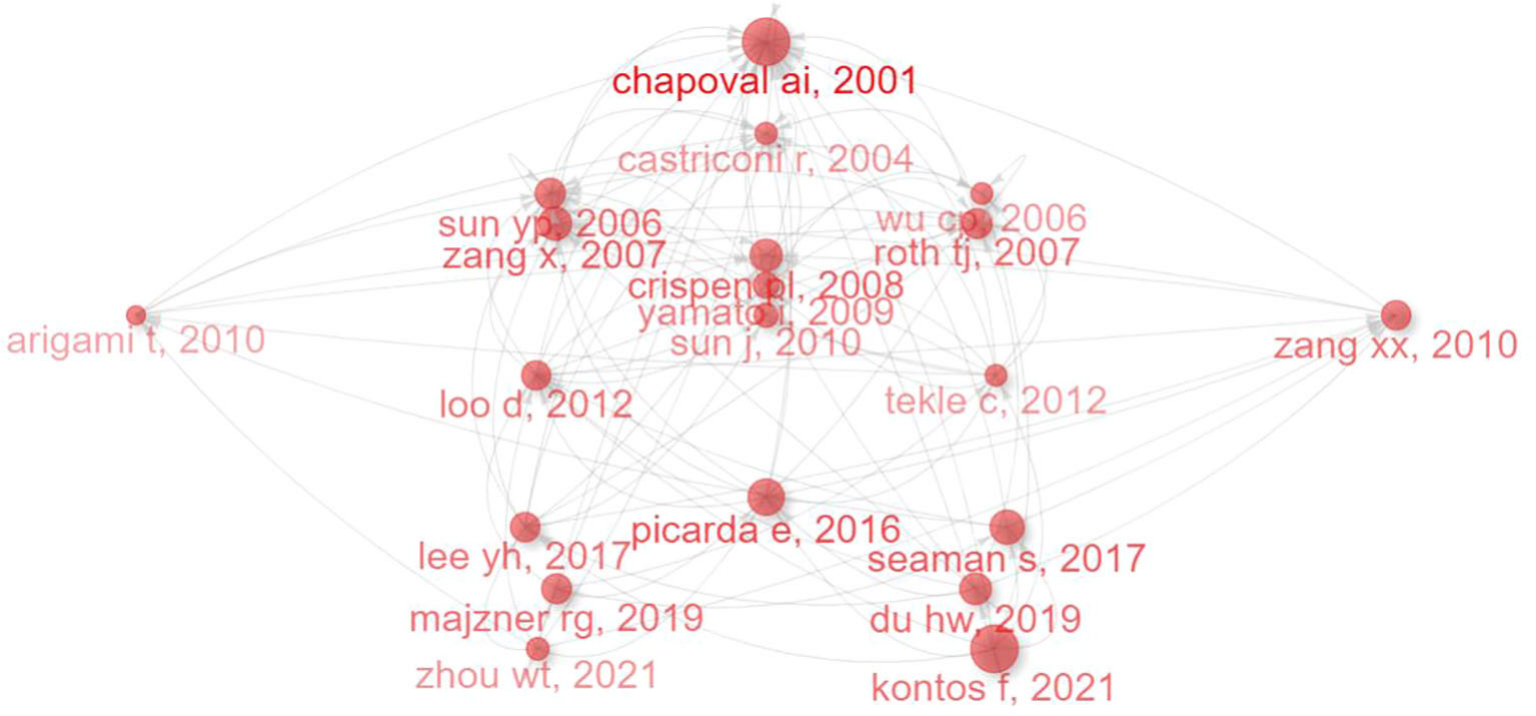
The evolution of CD276/B7-H3 research: a citation network analysis tracing the development from mechanistic discovery to immunotherapy.

**Figure 9** and **Table 7** present a citation network analysis (based on Web of Science data) of key publications on CD276/B7-H3, tracing the intellectual evolution of the field. Foundational studies from the early 2000s, such as Chapoval et al. (2001) on B7-H3’s costimulatory function and Castriconi et al. (2004) on its role in immune evasion, established core themes that spurred specialized research trajectories. These works prompted subsequent investigation into immunotherapy, receptor targeting, and clinical applications.

**Table 7:** The top 20 most influential publications on CD276 in oncology.

Title	Year	LCS	GCS
B7-H3: A COSTIMULATORY MOLECULE FOR T CELL ACTIVATION AND IFN-Γ PRODUCTION	2001	312	857
IDENTIFICATION OF 41G-B7-H3 AS A NEUROBLASTOMA-ASSOCIATED MOLECULE THAT EXERTS A PROTECTIVE ROLE FROM AN NK CELL-MEDIATED LYSIS	2004	80	238
B7-H3 AND B7-H4 EXPRESSION IN NON-SMALL-CELL LUNG CANCER	2006	114	292
RELATIONSHIP BETWEEN CO-STIMULATORY MOLECULE B7-H3 EXPRESSION AND GASTRIC CARCINOMA HISTOLOGY AND PROGNOSIS	2006	79	126
B7-H3 AND B7X ARE HIGHLY EXPRESSED IN HUMAN PROSTATE CANCER AND ASSOCIATED WITH DISEASE SPREAD AND POOR OUTCOME	2007	135	326
B7-H3 LIGAND EXPRESSION BY PROSTATE CANCER: A NOVEL MARKER OF PROGNOSIS AND POTENTIAL TARGET FOR THERAPY	2007	111	209
TUMOR CELL AND TUMOR VASCULATURE EXPRESSION OF B7-H3 PREDICT SURVIVAL IN CLEAR CELL RENAL CELL CARCINOMA	2008	126	232
CLINICAL IMPORTANCE OF B7-H3 EXPRESSION IN HUMAN PANCREATIC CANCER	2009	90	147
B7-H3 LIGAND EXPRESSION BY PRIMARY BREAST CANCER AND ASSOCIATED WITH REGIONAL NODAL METASTASIS	2010	77	124
CLINICAL SIGNIFICANCE AND REGULATION OF THE COSTIMULATORY MOLECULE B7-H3 IN HUMAN COLORECTAL CARCINOMA	2010	85	140
TUMOR ASSOCIATED ENDOTHELIAL EXPRESSION OF B7-H3 PREDICTS SURVIVAL IN OVARIAN CARCINOMAS	2010	107	207
DEVELOPMENT OF AN FC-ENHANCED ANTI-B7-H3 MONOCLONAL ANTIBODY WITH POTENT ANTITUMOR ACTIVITY	2012	106	216
B7-H3 CONTRIBUTES TO THE METASTATIC CAPACITY OF MELANOMA CELLS BY MODULATION OF KNOWN METASTASIS-ASSOCIATED GENES	2012	79	127
MOLECULAR PATHWAYS: TARGETING B7-H3 (CD276) FOR HUMAN CANCER IMMUNOTHERAPY	2016	163	406
INHIBITION OF THE B7-H3 IMMUNE CHECKPOINT LIMITS TUMOR GROWTH BY ENHANCING CYTOTOXIC LYMPHOCYTE FUNCTION	2017	108	249
ERADICATION OF TUMORS THROUGH SIMULTANEOUS ABLATION OF CD276/B7-H3-POSITIVE TUMOR CELLS AND TUMOR VASCULATURE	2017	142	305
CAR T CELLS TARGETING B7-H3, A PAN-CANCER ANTIGEN, DEMONSTRATE POTENT PRECLINICAL ACTIVITY AGAINST PEDIATRIC SOLID TUMORS AND BRAIN TUMORS	2019	111	455
ANTITUMOR RESPONSES IN THE ABSENCE OF TOXICITY IN SOLID TUMORS BY TARGETING B7-H3 VIA CHIMERIC ANTIGEN RECEPTOR T CELLS	2019	123	340
B7-H3/CD276: AN EMERGING CANCER IMMUNOTHERAPY	2021	81	203
B7-H3: AN ATTRACTIVE TARGET FOR ANTIBODY-BASED IMMUNOTHERAPY	2021	108	257

LCS, local citation score; GCS, Global Citation Score.

The network shows strong thematic clusters around mechanistic studies, prognostic significance, and emerging therapies. Key nodes such as Zang et al. (2007) bridge basic and clinical research, while recent works by Picarda et al. (2016) and Kontos et al. (2021) highlight the shift toward CAR-T cells and antibody-based treatments. The cohesion among clusters underscores a structured and cumulative knowledge base, illustrating a clear pathway from discovery to translational research and confirming B7-H3’s growing importance as a therapeutic target in oncology.

### 3.7 Keyword analysis and research trends

**Figure 10:**
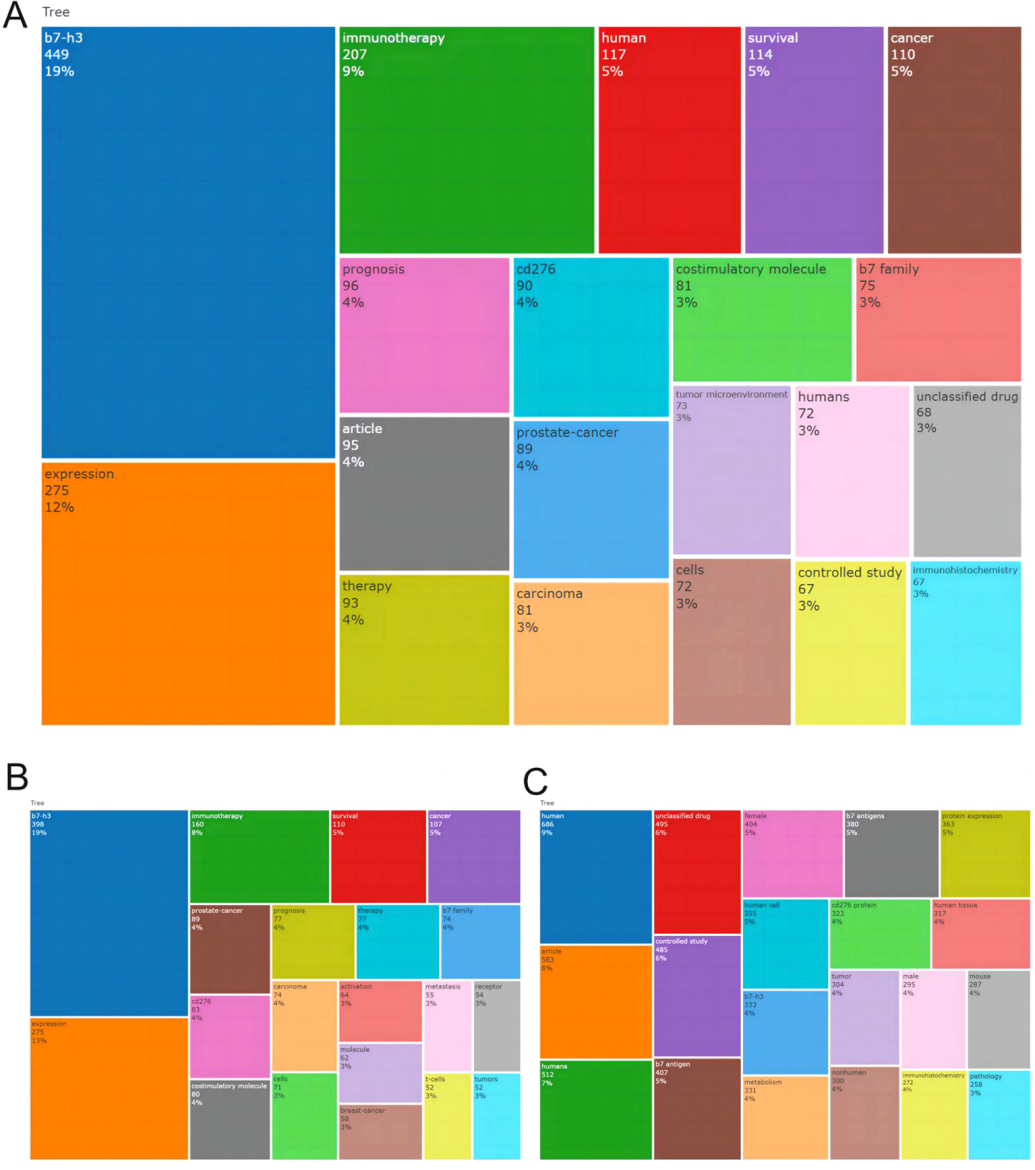
Treemap of the top 20 keywords in CD276/B7-H3 research. **(A)** Keyword treemap based on the merged dataset of WoS and Scopus. **(B)** Keyword treemap derived from Web of Science (WoS). **(C)** Keyword treemap derived from Scopus. The size of each block represents the frequency of the keyword, with percentages indicating the proportion relative to total keyword occurrences across all publications in each database.

Based on the keyword co-occurrence analysis derived from the merged, WoS, and Scopus datasets (**Figure 10**, showing the top 20 keywords from each database), the research landscape of CD276/B7-H3 is clearly anchored by two dominant themes. Across all three datasets, “b7-h3” emerged as the most frequent keyword (19% in merged), serving as the thematic core of the field. “Immunotherapy” ranked as the second most prominent theme (8–9% across datasets), underscoring the field’s strong translational orientation toward therapeutic applications.

The keyword landscape reveals a tripartite structure of research focus. First, cancer-type-specific studies are represented by terms such as “prostate-cancer” (4%) and “carcinoma” (5%), indicating concentrated efforts in specific malignancies. Second, mechanistic and biological investigations are reflected by keywords including “expression” (12% in merged), “tumor microenvironment” (3%), “cells,” and “receptor” (2–3%), which represent ongoing basic science inquiries into B7-H3 function. Third, clinical and translational research is captured by terms such as “therapy” (4%), “prognosis” (4%), “metastasis” (3%), and “human”/”humans” (8% combined), highlighting the field’s commitment to understanding B7-H3’s clinical implications and advancing toward patient applications.

**Figure 11:**
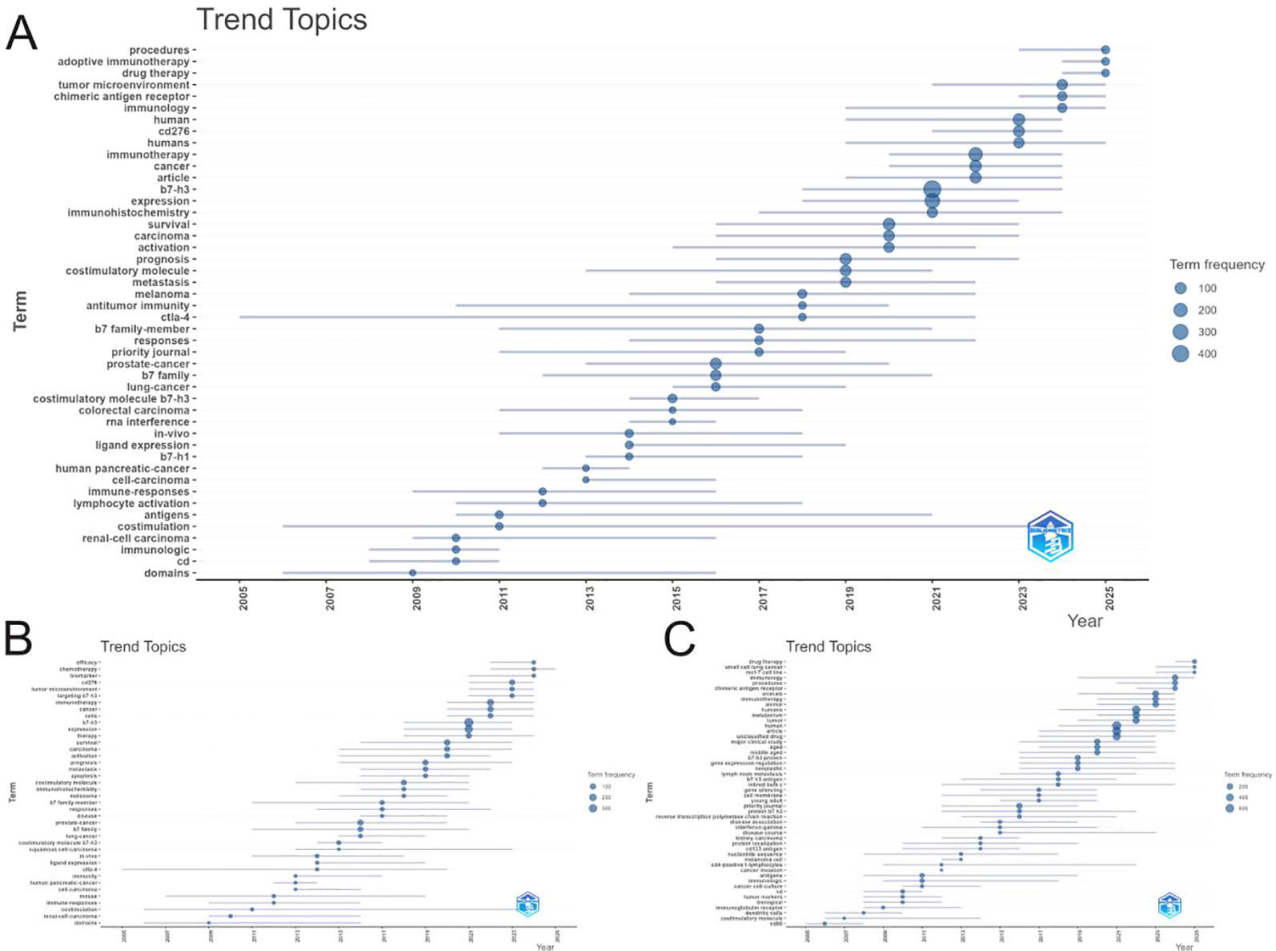
Trend topics analysis of CD276/B7-H3 research. **(A)** Trend topics based on the merged dataset of WoS and Scopus. **(B)** Trend topics derived from WoS. **(C)** Trend topics derived from Scopus. The horizontal axis represents the time line (publication year), and the vertical axis lists the keywords. The bubble size indicates the frequency of the keyword, and the position reflects the median year of occurrence.

The temporal evolution of CD276/B7-H3 research exhibits a clear and logical progression(**Figure 11**). Early research phases (pre-2010) were characterized by foundational terms such as “costimulation,” “domains,” and “immune-responses,” alongside model system keywords like “mouse” and specific antigens, reflecting investigations into the basic molecular and immunologic functions of B7-H3. From approximately 2010 to 2018, the field shifted toward disease-specific and prognostic studies, with rising focus on cancers such as renal-cell carcinoma, prostate-cancer, lung-cancer, and melanoma. Keywords including “prognosis,” “metastasis,” and “survival” grew in prominence, reflecting increased interest in the clinical and pathological significance of B7-H3 expression. Since around 2018, research has become increasingly dominated by translational and therapeutic themes. The most frequent and recently emerging terms are “immunotherapy,” “tumor microenvironment,” and “chimeric antigen receptor.” Notably, “CAR-T” and “targeting B7-H3” exhibit the most recent median years (2023–2024), highlighting the current emphasis on molecularly targeted immunotherapies.

This keyword trajectory illustrates a coherent and disease-relevant evolution, marking the progression of CD276/B7-H3 from a novel immune molecule to a promising therapeutic target, with strong and growing research interest in clinical applications. The consistency of keyword patterns across all three datasets further validates the robustness of these observed trends.

### 3.8 Alignment of research focus with CD276 pan-cancer expression profile

**Figure 12:**
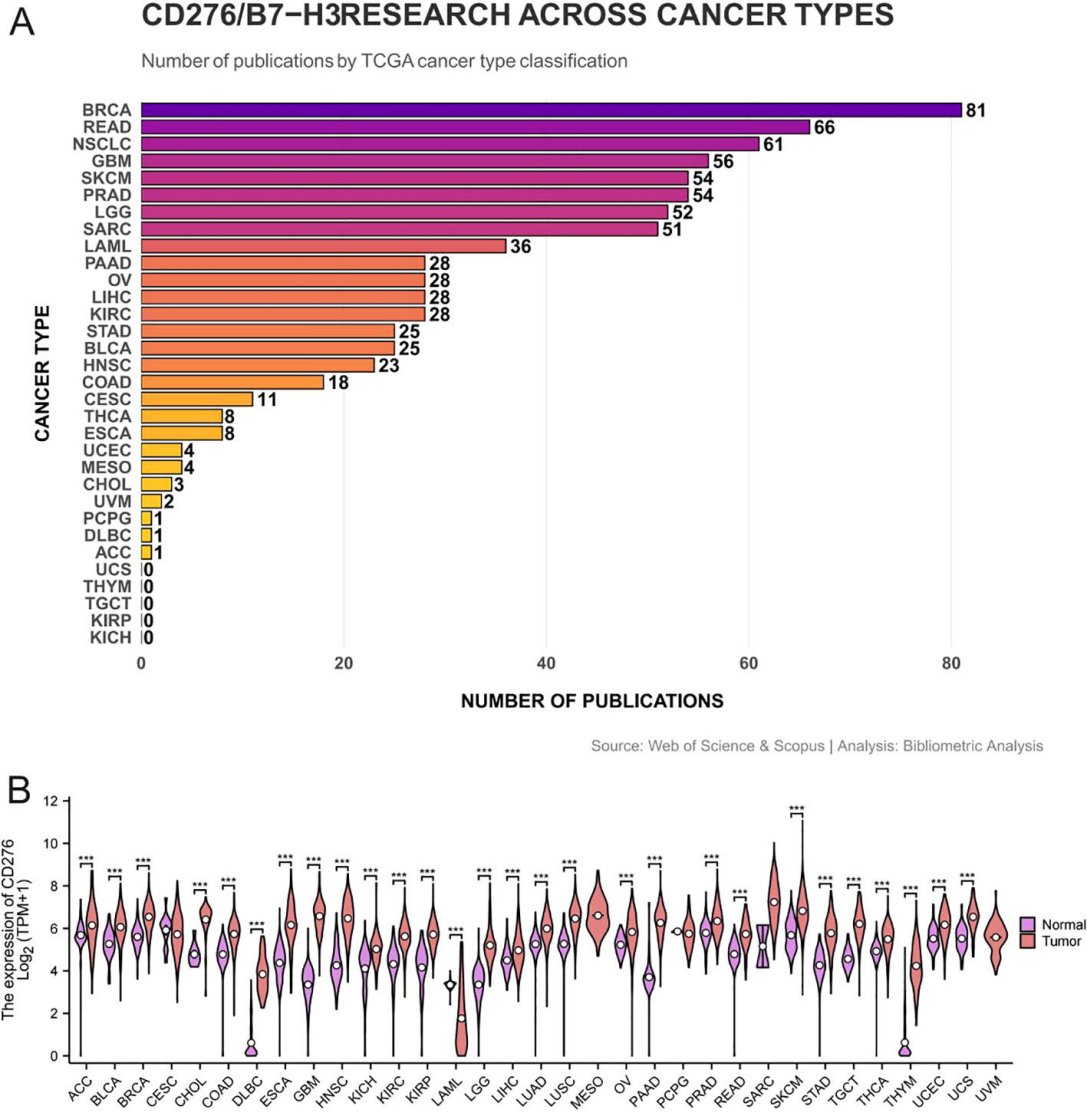
Comparison of research output and CD276/B7-H3 expression across cancer types. Bar graphs show the number of publications (research output) for each cancer type. The heatmap represents CD276 mRNA expression levels (log2-transformed RSEM values) from The Cancer Genome Atlas (TCGA) across 33 cancer types. Cancer type abbreviations are defined in the table below.

To further contextualize the bibliometric findings, we compared the research output on CD276/B7-H3 across cancer types with both transcriptomic data reflecting CD276 expression levels and global cancer incidence rates (**Figure 12**). This integrative analysis reveals several important alignments and divergences between molecular relevance, disease burden, and scientific attention.

Cancers exhibiting both high CD276 expression and substantial research coverage include breast cancer (BRCA), non-small cell lung cancer (NSCLC), glioblastoma (GBM), and prostate adenocarcinoma (PRAD). These alignments suggest that research efforts are well-justified on both molecular and epidemiological grounds.

However, several cancers with notable CD276 upregulation and high incidence rates—such as stomach adenocarcinoma (STAD), liver hepatocellular carcinoma (LIHC), and colorectal cancer (COAD/READ)—received relatively moderate research attention. This discrepancy suggests that these malignancies may be under-investigated given their molecular characteristics and public health burden.

A notable exception is acute myeloid leukemia (LAML), which has attracted considerable research output (36 publications) despite inconsistent reports regarding CD276 expression. This may reflect the clinical urgency of LAML and the pursuit of novel therapeutic targets, underscoring that research prioritization is not driven solely by expression levels but also by factors such as aggressiveness and unmet medical needs.

While the TCGA-based expression analysis in **Figure 12** is limited to adult tumors, B7-H3 plays a significant role in pediatric oncology. The molecule was originally identified as a tumor-associated antigen in neuroblastoma, the most common extracranial solid tumor in children (27). Subsequent studies have documented high B7-H3 expression in multiple pediatric solid malignancies, including neuroblastoma (>95% positive), medulloblastoma (~95% positive), Ewing sarcoma, and osteosarcoma, with expression frequently associated with metastatic progression and poor prognosis (43). In pediatric brain tumors, B7-H3 expression is common but exhibits inter-tumoral heterogeneity; for example, approximately 30% of diffuse midline gliomas show minimal or no expression (57). Notably, the seminal study by Majzner et al. (2019) (48) demonstrated potent preclinical activity of B7-H3 CAR T cells against pediatric solid and brain tumors, and early-phase clinical trials are currently underway (58). These pediatric findings complement the adult-focused analysis in **Figure 12** and underscore B7-H3’s importance as a pan-cancer therapeutic target across all age groups.

Overall, the distribution of publications reflects a research landscape shaped by a combination of molecular biology, epidemiology, and clinical imperatives. These observations highlight opportunities to further optimize research resource allocation to better align with both biological significance and population health impact.

## 4 Discussion

This study presents the first integrated bibliometric analysis of global research on CD276/B7-H3 in oncology over the past quarter-century (2001–2025). By systematically merging data from Web of Science and Scopus and employing multiple analytical tools, we have not only confirmed the field’s explosive growth but also quantitatively mapped its intellectual structure, collaborative networks, and thematic evolution. Unlike previous narrative reviews that have qualitatively summarized the biological functions and therapeutic potential of B7-H3 (17, 25, 44), our work provides a data-driven macroscopic perspective that objectively delineates the knowledge landscape, identifies pivotal contributions, and, for the first time, benchmarks research activity against the pan-cancer expression profile of CD276. This approach offers a complementary and strategic framework for understanding the field’s trajectory and guiding future investigations.

### 4.1 Exponential growth and geographic disparities in research output and impact

The field of CD276/B7-H3 cancer research has experienced remarkable growth, with an annual publication increase of 21.77%, reflecting the molecule’s ascendance as a critical immune checkpoint and therapeutic target. This trajectory mirrors the broader shift in immuno-oncology beyond canonical checkpoints like PD-1/PD-L1 and CTLA-4 (8, 9). While China and the United States emerged as the dominant contributors in terms of raw output (accounting for over 70% of publications), a striking disparity exists between productivity and impact. Chinese institutions, particularly Soochow University and Peking University, lead in publication volume but exhibit relatively low average citations per paper (AC ≈ 27), whereas the United States and several European countries (e.g., France, Norway, Sweden) demonstrate substantially higher per-article influence (AC > 60). This discrepancy may reflect differences in research emphasis (e.g., more mechanistic/translational studies vs. confirmatory work), language barriers, or varying levels of international integration. The persistently strong China–U.S. collaborative axis identified in our network analysis underscores a vital channel for knowledge exchange, yet the overall low international co-authorship rate (13.25%) signals untapped potential for broader global cooperation, which could enhance the visibility and impact of research from less-connected regions.

### 4.2 Thematic evolution: from molecular characterization to clinical translation

Keyword trajectory analysis reveals a clear and logical progression in CD276 research. The early phase (pre-2010) was dominated by fundamental immunology concepts—”costimulation,” “B7 family,” “expression”—establishing the basic biology of B7-H3 (23). However, the understanding of B7-H3’s immunological role has undergone a significant evolution. While initially characterized as a T-cell costimulator, subsequent studies revealed a more complex functional spectrum. Prasad et al. first demonstrated that B7-H3 expressed on dendritic cells acts as a negative regulator of T-cell activation, preferentially down-regulating Th1-mediated immune responses (26). This inhibitory function was further supported by Castriconi et al., who identified 4Ig-B7-H3 as a tumor-associated antigen in neuroblastoma that protects tumor cells from natural killer (NK) cell-mediated lysis (27). These seminal findings established B7-H3 as a bifunctional immune molecule with both co-stimulatory and co-inhibitory properties, fundamentally reshaping its classification from a costimulator to an immune checkpoint (29, 59).This was followed by a transitional period (2010–2018) marked by disease-specific studies and prognostic assessments across multiple cancer types, including prostate, lung, renal, and breast carcinomas (31, 34, 37, 42, 60). The most recent phase (post-2018) is characterized by a decisive shift toward therapeutic applications, with “immunotherapy,” “CAR-T,” “tumor microenvironment,” and “bispecific antibody” emerging as dominant themes. This evolution is corroborated by the high citation impact of recent publications on B7-H3-targeting CAR T-cell therapies (48, 61) and antibody-drug conjugates (19, 44), indicating that the field is rapidly transitioning from mechanistic understanding to clinical innovation. The dual-map overlay further supports this interpretation, illustrating robust knowledge flows from foundational molecular/immunological sciences to applied clinical research, underscoring the interdisciplinary nature of the field.

### 4.3 Alignment of research effort with molecular and epidemiological burden: a call for strategic rebalancing

A unique contribution of this study is the integrative analysis of research output against CD276 transcriptomic expression and cancer incidence (**Figure 12**). While research focus aligns well with high-expression/high-incidence cancers such as breast, lung, and prostate malignancies, several notable under-investigated cancers emerge. Stomach adenocarcinoma (STAD), liver hepatocellular carcinoma (LIHC), and colorectal cancer (COAD/READ) exhibit both elevated CD276 expression and substantial global incidence, yet they have attracted relatively modest research attention. This mismatch suggests that current research priorities may not fully reflect either the molecular relevance or the public health burden of these malignancies. Conversely, acute myeloid leukemia (LAML) has garnered considerable interest despite inconsistent reports of CD276 expression, likely driven by the clinical urgency of the disease and the pursuit of novel therapeutic options. These findings highlight an opportunity to strategically reallocate research resources toward cancers where CD276 targeting could have maximal clinical impact, particularly gastrointestinal and hepatic tumors. Future studies should prioritize validating B7-H3 as a therapeutic target in these contexts and accelerating preclinical development.

### 4.4 Implications for future research and clinical translation

Building on our findings, we propose several strategic directions for the field:

Deepening mechanistic understanding: Despite the therapeutic focus, the context-dependent mechanisms of B7-H3 in immune suppression, therapy resistance, and stromal crosstalk remain incompletely understood (20, 24, 28). Future work should elucidate these pathways to inform rational combination strategies.

Accelerating clinical translation: While CAR-T cells and ADCs targeting B7-H3 have shown preclinical promise (19, 48), clinical data remain nascent. Larger trials, combination regimens (e.g., with PD-1 inhibitors or chemotherapy), and next-generation modalities (e.g., bispecific T-cell engagers) are urgently needed.

Fostering international collaboration: The low rate of international co-authorship calls for initiatives to promote cross-border partnerships, particularly between high-output countries (China, U.S.) and regions with emerging research capacity.

Addressing research disparities: Funding agencies and researchers should consider the misalignment identified in **Figure 12** and prioritize under-studied cancers with high CD276 expression, such as gastric, liver, and colorectal cancers.

Integrating multi-omics data: As proteomic and spatial transcriptomic data become more accessible, future bibliometric analyses should incorporate protein-level expression to refine target prioritization.

### 4.5 Limitations

This study has several limitations. First, while we combined two major databases, the exclusion of PubMed may have omitted some relevant literature, particularly from regional journals. Second, despite rigorous data cleaning, variations in author and institution naming could introduce minor inaccuracies in network analyses. Third, bibliometrics is inherently quantitative; it does not capture the qualitative nuances of research quality or experimental design. Finally, the pan-cancer expression analysis in this study was primarily based on transcriptomic data from The Cancer Genome Atlas (TCGA). While this provides broad coverage across cancer types, mRNA expression does not always correlate perfectly with protein abundance due to post-transcriptional regulation. Although we attempted to incorporate proteomic data from resources such as CPTAC, the limited number of cancer types with available protein-level data (n = 10) precluded a comprehensive pan-cancer proteomic analysis. As public proteomic databases expand, future studies should aim to integrate protein expression data to further validate the alignment between research focus and molecular target relevance.

## 5 Conclusion

In conclusion, this study offers the first holistic bibliometric overview of the global research landscape on CD276/B7-H3 in cancer. The field has evolved substantially from initial molecular and functional characterization to an emphasis on targeted immunotherapies and clinical applications. China and the U.S. lead in research output, whereas European and American institutions demonstrate high per-article influence, underscoring the importance of both productivity and impact. Thematic progression—from basic expression studies to immunotherapy and tumor microenvironment interactions—illustrates a dynamic and translational research trajectory.

Looking forward, we recommend that future research prioritize the following areas:

Deepening the understanding of context-specific mechanisms of B7-H3 in immune suppression and treatment resistance; Accelerating clinical evaluation of emerging modalities, such as next-generation CAR T-cells, bispecific antibodies, and combination therapies; Fostering international and interdisciplinary collaborations to integrate real-world data and support personalized treatment strategies; Addressing disparities between research focus and disease burden, particularly in gastrointestinal and hepatic cancers with high CD276 expression.

With continued innovation and collaboration, CD276/B7-H3 is poised to remain a central target in cancer immunotherapy, offering new avenues for overcoming treatment resistance and improving patient outcomes.

## Data Availability

The original contributions presented in the study are included in the article/**Supplementary Material**. Further inquiries can be directed to the corresponding authors.
