# Supplementary figures and images for: From mechanism to clinical: research evolution and hotspot analysis of CD276/B7-H3 in cancer immunotherapy
Source: Front Immunol. 2026 Apr 1;17:1751400. doi: 10.3389/fimmu.2026.1751400 (PMC13079588; doi:10.3389/fimmu.2026.1751400)

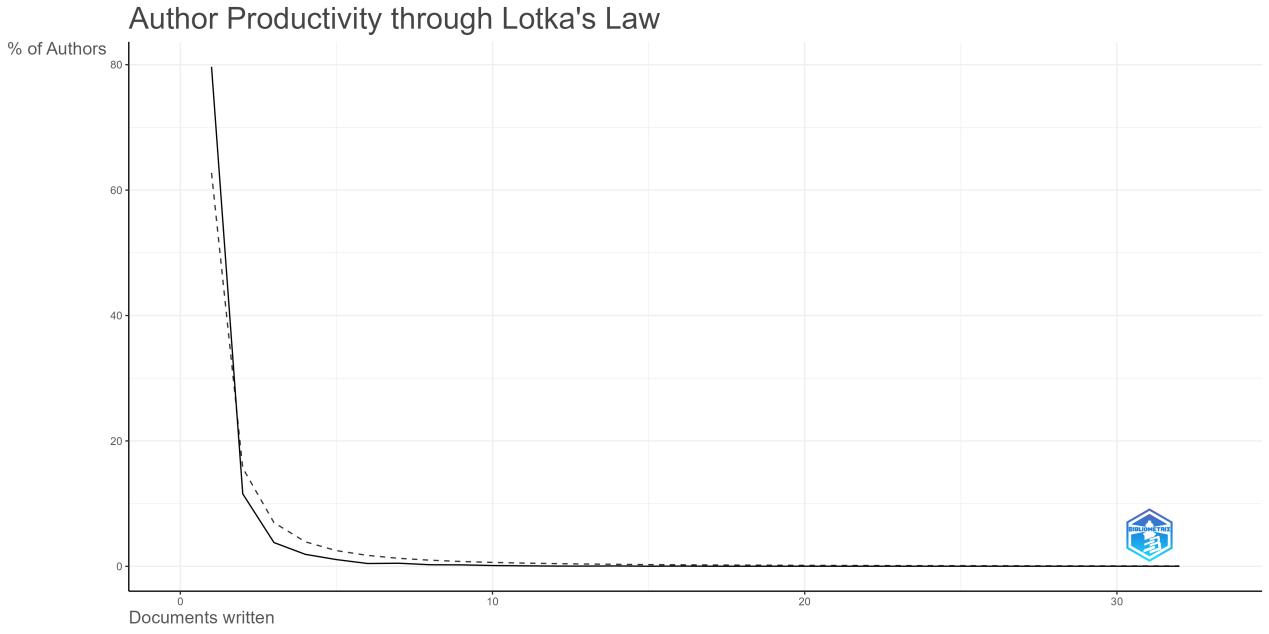

Supplement: Supplementary file 1 [file Image1.jpeg]

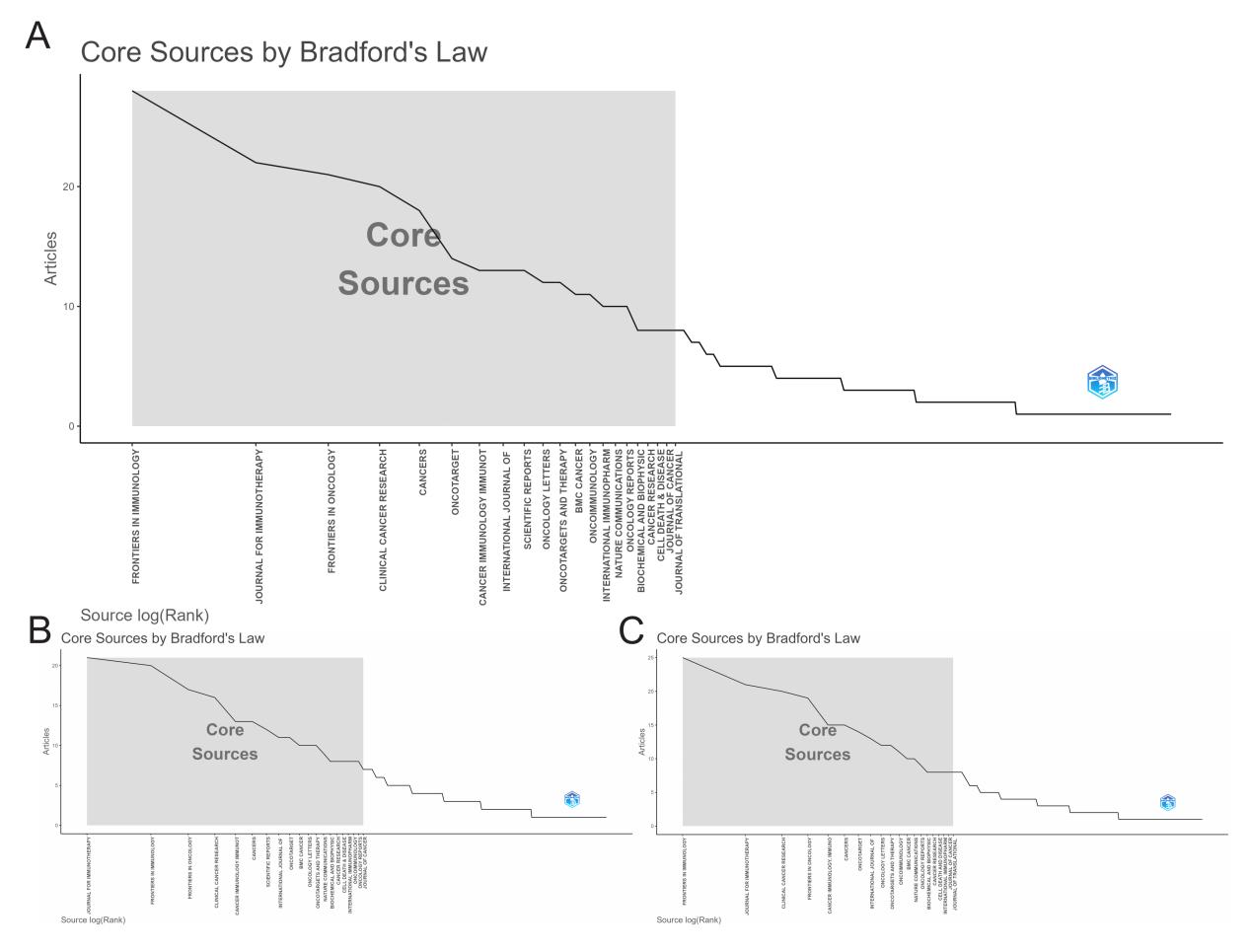

Supplement: Supplementary file 2 [file Image2.jpeg]
